# *Helicobacter pylori* eradication for primary prevention of gastric cancer: progresses and challenges

**DOI:** 10.1016/j.jncc.2024.06.006

**Published:** 2024-07-20

**Authors:** Zongchao Liu, Hengmin Xu, Weicheng You, Kaifeng Pan, Wenqing Li

**Affiliations:** 1 State Key Laboratory of Holistic Integrative Management of Gastrointestinal Cancers, Beijing Key Laboratory of Carcinogenesis and Translational Research, Department of Cancer Epidemiology, Peking University Cancer Hospital & Institute, Beijing, China; 2 Key Laboratory of Carcinogenesis and Translational Research (Ministry of Education/Beijing), Department of Cancer Epidemiology, Peking University Cancer Hospital and Institute, Beijing, China

**Keywords:** Gastric cancer, *Helicobacter pylori*, Intervention trial, Cost-effectiveness, Precision prevention

## Abstract

Gastric cancer remains a significant global health challenge, causing a substantial number of cancer-related deaths, particularly in China. While the exact causes of gastric cancer are still being investigated, *Helicobacter pylori* (*H. pylori*) infection has been identified as the primary risk factor, which triggers chronic inflammation and a multistage progression of gastric lesions that may lead to carcinogenesis over a long latency time. Since the 1990s, numerous efforts have focused on assessing the effectiveness of *H. pylori* eradication in preventing new cases of gastric cancer among both the general population and patients who have undergone early-stage cancer treatment. This body of work, including several community-based interventions and meta-analyses, has shown a reduction in both the incidence of and mortality from gastric cancer following *H. pylori* treatment, alongside a decreased risk of metachronous gastric cancer. In this review, we seek to consolidate current knowledge on the effects of *H. pylori* treatment on gastric cancer prevention, its systemic consequences, cost-effectiveness, and the influence of antibiotic resistance and host characteristics on treatment outcomes. We further discuss the potential for precision primary prevention of *H. pylori* treatment and comment on the efficient implementation of test-and-treat policies and allocation of health resources towards minimizing the burden of gastric cancer globally.

## 1 Introduction

Gastric cancer (GC) is still a major public health concern, ranking as one of the most prevalent cancers and top leading causes of cancer-related deaths worldwide.^1^ The burden disproportionately falls on populations in East Asia, Latin America, and eastern Europe, with almost half of new cases and related deaths occurring in China. Although the etiology of GC remains to be elucidated, *Helicobacter pylori* (*H. pylori*) infection has been established as the most important risk factor,^2^ responsible for over 70% of all GCs.^3^ Approximately 79% of non-cardia GC in Asia and 87% in Europe and North America, along with 62% of cardia GC in Asia, could be attributable to *H. pylori* infection.^4^ Known as a gram-negative bacterium that infects more than 40% of the world's population,^5^ *H. pylori* was the first bacterium to be classified as a Group 1 carcinogen by the International Agency for Research on Cancer (IARC), which induces chronic inflammation of gastric mucosa and multistage progression of gastric lesions and ultimately, over a long latency time, leads to gastric carcinogenesis.^6^^,^^7^ The latest comprehensive synthesis of evidence reveals a declining trend of GC incidence in relation to the declining trend of *H. pylori* prevalence globally and in various countries,^5^ supporting the hypothesis that reduction of *H. pylori* prevalence may reduce the risk of GC across populations.

Since 1990s, considerable efforts have been made to figure out the efficacy and effectiveness of *H. pylori* treatment on the prevention of new GC for healthy population^8^, ^9^, ^10^, ^11^, ^12^, ^13^, ^14^, ^15^, ^16^, ^17^, ^18^, ^19^, ^20^, ^21^, ^22^ or patients with early GC undergoing endoscopic mucosal resection.^23^, ^24^, ^25^, ^26^ As previous randomized controlled trials (RCTs) were conducted with a modest scale, a limited sample size has precluded the possibility of clarifying the full consequences of *H. pylori* treatment. Our team therefore conducted the Mass Intervention Trial in Linqu, Shandong Province (MITS), the largest-to-date community-based trial that involved a total of over 180,000 participants.^27^ Linqu County in Shandong Province is a known high-risk area for GC in China with an age-standardized incidence rate of 48.98/10^5^ and mortality rate of 30.99/10^5^ from 2012 to 2019.^28^ While the MITS is expected to eventually answer the effectiveness of introducing large population-based *H. pylori* treatment programs in community-based settings,^27^ data also needs to be accumulated to identify the subgroup of individuals that would benefit the most from *H. pylori* treatment. Here we summarize the available data on the effects of *H. pylori* treatment on GC, and its systemic consequences and cost-effectiveness, also reviewing evidence on elucidating the impact of antibiotic resistance and host characteristics on eradication failure. We further comment on the opportunities of fulfilling precision primary prevention of GC, aiming to provide insights into the test-and-treat policy implementation and allocation of health resources.

## 2 Effect of *H. pylori* treatment in reducing GC risk

**Fig. 1:**
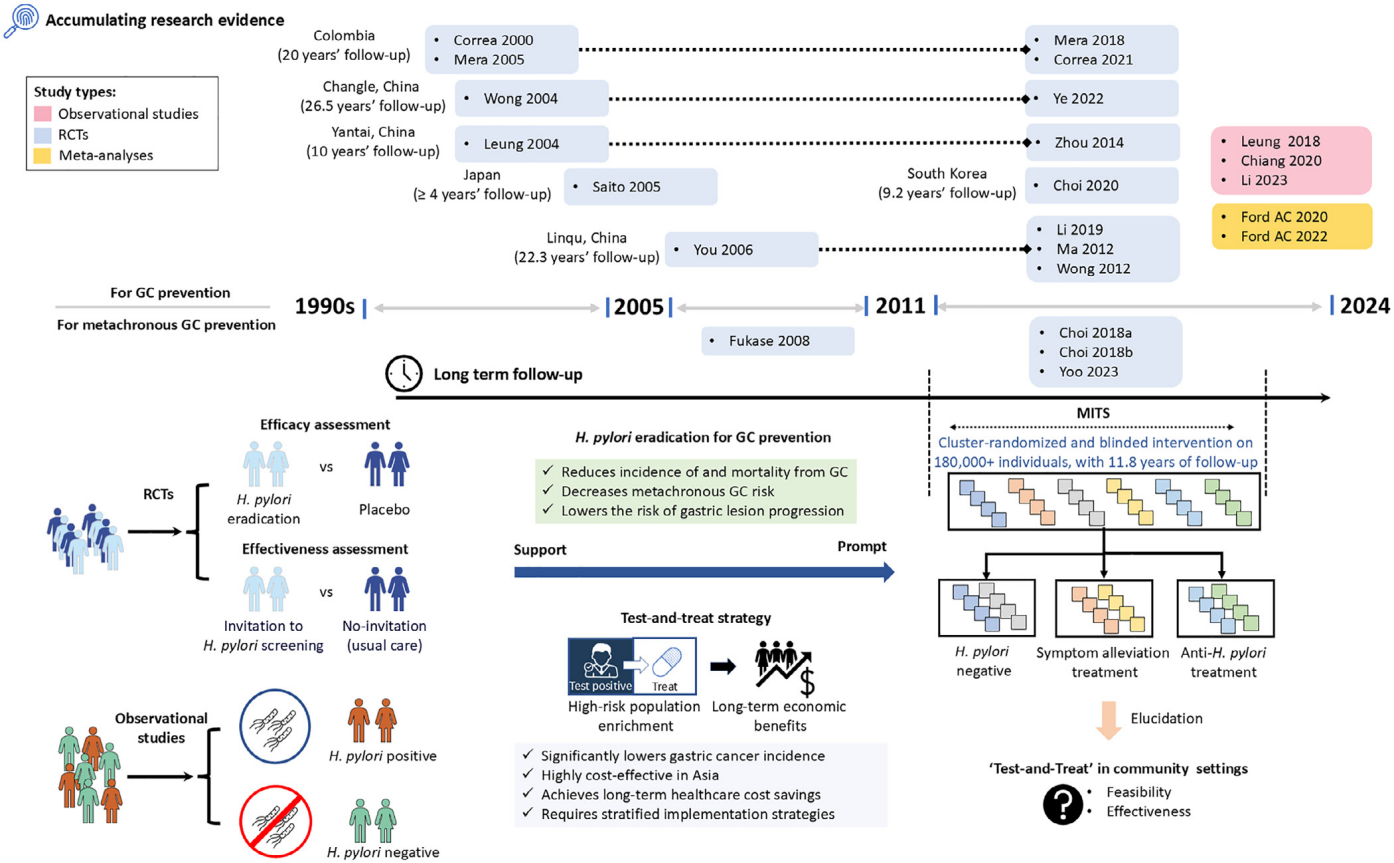
Research trajectory and implications of *H. pylori* eradication for gastric cancer prevention. The timeline compiles evidence from randomized controlled trials (RCTs) and observational studies, confirming that *H. pylori* eradication diminishes both GC incidence and mortality, and lowers metachronous GC risk. RCTs comparing *H. pylori* eradication to placebo or no intervention for GC prevention are categorized by trial locations that were highlighted by the map. The related trial studies are annotated with the longest follow-up durations, as reported in the most recent research. The necessity for tailored treatment approaches in GC prevention has been highlighted, and studies with long-term follow-up affirm the ‘test-and-treat’ strategy's effectiveness and cost-benefit, especially in high-risk Asian populations. The MITS trial, with its community-based nature, long-term follow-up and unique large sample size, further explores the feasibility of the ‘test-and-treat’ strategy, offering opportunities to develop more nuanced, personalized intervention strategies. GC, gastric cancer; *H. pylori, Helicobacter pylori*; MITS, Mass Intervention Trial in Shandong; RCT, randomized controlled trial.

Numerous studies have been conducted worldwide to assess the impact of *H. pylori* treatment on the prevention of GC among healthy populations, with notable trials carried out in Colombia, Japan, Korea, and various high-risk regions in China, including Changle County in Fujian Province, and Yantai City and Linqu County in Shandong Province (Table 1, Fig. 1).^8^, ^9^, ^10^, ^11^, ^12^, ^13^, ^14^, ^15^, ^16^, ^17^, ^18^, ^19^, ^20^, ^21^, ^22^ The Shandong Intervention Trial (SIT, NCT0033976; https://clinicaltrials.gov/) that our team conducted in Linqu County was recognized as the first to demonstrate a statistically significant decrease in GC incidence from *H. pylori* treatment during 14.7 years’ follow-up (1995–2010, odds ratio [OR] = 0.61, 95% confidence interval [CI]: 0.38–0.96). The benefit on GC incidence persisted during 22.3 years of follow-up (1995–2019, OR = 0.48, 95% CI: 0.32–0.71) and further conferred a marked reduction in GC mortality (hazard ratio [HR] = 0.62, 95% CI: 0.39–0.99). Further evidence from the SIT underlined the benefits of *H. pylori* eradication in preventing GC in subjects with mild and severe gastric lesions.^18^, ^19^, ^20^ This was echoed by the RCT with a 26.5-year follow-up (1994–2020) from Changle District of Fujian Province, although this trial emphasized the protective effects primarily in individuals without precancerous gastric lesions at the baseline.^13^ In addition, a trial (2004–2018) in Korea demonstrated that *H. pylori* eradication reduced the risk of GC in individuals with a family history (HR = 0.45, 95% CI: 0.21–0.94).^21^ Incorporating existing trials, meta-analyses substantiate the beneficial effect of *H. pylori* treatment on preventing GC (risk ratio [RR] = 0.54, 95% CI: 0.41–0.72).^29^^,^ ^30^

**Table 1:** RCTs of *H. pylori* treatment versus placebo or no treatment in GC prevention in healthy individuals.

Study	Location	Follow-up, years	*H. pylori* eradication therapy regimen	Pre-neoplastic lesions at baseline, %	Eradication rate^c^, %	Mean age at baseline (years, range)	Sample size (GC cases)		Effect estimates (95% CI) e
							Treatment	Placebo/control	
Correa 2000, Mera 2005, Mera 2018 and Correa 2021^8^, ^9^, ^10^, ^11^	Two communities in Narino Province, Colombia	20	Bismuth subsalicylate 262 mg, amoxicillin 500 mg and metronidazole 375 mg t.i.d. for 2 weeks.	100.0^a^	58.0	51 (29–69)	437 (3)^d^	415 (2)	EE1: 1.42 (0.24–8.48)
Wong 2004 and Ye 2022^12^^,^^13^	Seven villages in Changle County, Fujian Province, China	26.5	Omeprazole 20 mg, coamoxiclav 750 mg and metronidazole 400 mg b.i.d. for 2 weeks.	37.7^a^	83.7	42 (35–65)	817 (21)	813 (35)	EE1: 0.60 (0.35–1.02), EE2: 0.57 (0.33–0.98)
Saito 2005^14^	145 centres in Japan	≥4	Lansoprazole 30 mg, amoxicillin 1.5 g and clarithromycin 400 mg o.d. for 1 week.	NR	74.4	NR (20–59)	379 (2)	313 (3)	EE1: 0.55 (0.09–3.27)
Wong 2012^15^	Twelve villages in Linqu County, Shandong Province, China	5	Omeprazole 20 mg, amoxicillin 1 g and clarithromycin 500 mg b.i.d. for 1 week.	100.0^a^	63.5	53 (35–64)	255 (3)	258 (1)	EE1: 3.04 (0.32–28.99)
Leung 2004 and Zhou 2014^16^^,^^17^	Eleven villages in Yantai City, Shandong Province, China	10	Omeprazole 20 mg, amoxicillin 1 g and clarithromycin 500 mg b.i.d. for 1 week.	45.5^b^	55.6	52 (35–75)	276 (2)	276 (7)	EE1: 0.29 (0.06–1.36)
You 2006, Ma 2012 and Li 2019^18^, ^19^, ^20^	Thirteen villages in Linqu County, Shandong Province, China	22.3	Omeprazole 20 mg and amoxicillin 1 g b.i.d. for 2 weeks.	98.5^a^	73.2	47 (35–64)	1130 (41)	1128 (78)	EE1: 0.52 (0.36–0.76), EE2: 0.48 (0.32–0.71)
Choi 2020^21^	One hospital in Goyang, South Korea	9.2	Lansoprazole 30 mg, amoxicillin 1 g and clarithromycin 500 mg b.i.d. for 1 week.	NR	60.4	48 (40–65)	912 (10)	914 (23)	EE1: 0.44 (0.21–0.91), EE2: 0.45 (0.21–0.94)
Pan-Li 2024^22^	980 villages in all 10 townships of Linqu County, Shandong Province, China	11.8	Omeprazole 20 mg b.i.d., tetracycline 750 mg t.i.d., metronidazole 400 mg t.i.d., and bismuth citrate 300 mg b.i.d. for 10 days.	NR	72.9	43 (25–54)	52,026 (354)	50,304 (399)	EE1: 0.86 (0.74–0.99), EE2: 0.87 (0.75–1.00)

Abbreviations: b.i.d., two times per day; CI, confidence interval; GC, gastric cancer; *H. pylori, Helicobacter pylori*; NR, not reported; o.d., one time per day; RCT, randomized controlled trial; t.i.d., three times per day.

a Defined as gastric atrophy, intestinal metaplasia or dysplasia.

b Defined as gastric atrophy or intestinal metaplasia, calculated based on Leung et al, 2004 (*n* = 435).

c True intention-to-treat analysis, with all drop-outs assumed to have failed eradication therapy.

d Gastric cancer cases reported in the treatment/placebo group and effect estimates in the Correa cohort were only available before 2005.

e Effect estimates for the risk of gastric cancer were derived from univariate analyses (effect estimate 1, EE1) and, if applicable, from multivariate analyses (effect estimate 2, EE2).

To eventually clarify the feasibility of enhancing the strategies for *H. pylori* eradication in community-based populations, our team initiated the MITS (ChiCTR-TRC-10,000,979; https://www.chictr.org.cn), a cluster-randomized, blinded, mass intervention trial enrolling a total of 980 villages inclusive of 180,284 eligible individuals aged 25–54 years in Linqu County, Shandong Province, China,^27^ which the international community expect would eventually conclude on the efficacy and effectiveness of implementing mass *H. pylori* screening and treatment as a public health policy (Fig. 1).^31^ Based on the block stratified cluster randomization scheme across 980 villages, individuals who were tested positive for *H. pylori* using a ^13^C-urea breath test (UBT) received 10-day quadruple anti-*H. pylori* treatment (initially called “high-dose therapy”^27^) or symptom alleviation treatment with a single dosage of omeprazole and bismuth citrate (initially called “low-dose therapy”^27^). The overall successful eradication rate was 72.9% (32,325 out of 44,329 with known 2nd C-UBT results) in participants receiving anti-*H. pylori* treatment, and 15.1% of participants receiving symptom alleviation treatment were *H. pylori* negative post-treatment. *H. pylori*-negative individuals did not receive any treatment. All participants were prospectively followed by Dec. 31, 2022. During 11.8 years’ follow-up (2011–2022), 1035 incident GC cases were documented. Based on the intention-to-treat analyses, individuals receiving anti-*H. pylori* therapy had a statistically significant reduction in GC incidence compared with those receiving symptom alleviation treatment (HR = 0.86, 95% CI: 0.74–0.99), with stronger effect observed for those having successful *H. pylori* eradication (HR = 0.81, 95% CI: 0.69–0.96) than failed treatment. The beneficial effect of successful eradication was particularly noteworthy for individuals aged 25–45 years.^22^

In addition to RCTs, observational studies based on real-world settings also have associated *H. pylori* treatment with a lower risk of GC. In a study from Hong Kong, China comparing databases of public hospitals with cancer registries, a statistically significant reduction in the risk of GC (standardized incidence ratio [SIR] = 0.42, 95% CI: 0.42–0.84) was observed in individuals aged 60 years and older who had received *H. pylori* treatment 10 or more years prior, highlighting the long-term protective effect of *H. pylori* treatment against GC in older populations.^32^ Another observational study in Matsu Islands, Chinese Taiwan, reported a significant reduction in occurrence of atrophic gastritis, intestinal metaplasia, and GC (risk reduction = 53%, 95% CI: 30%–69%), with no increase in the likelihood of adverse consequences.^33^ Additionally, in a retrospective study focusing on a diverse community-based population across the United States, *H. pylori*-infected individuals who received eradication therapy showed a significant reduction in the risk of developing non-cardia GC over eight years (HR = 0.37, 95% CI: 0.14–0.97) compared to untreated individuals.^34^

*H. pylori* treatment also leads to significantly decreased risk of subsequent (metachronous) GC for individuals with early GC undergoing endoscopic mucosal resection (Table 2).^23^, ^24^, ^25^, ^26^ Choi et al. found that patients with early GC who received *H. pylori* treatment had a lower risk of metachronous GC (HR = 0.50, 95% CI: 0.26–0.94) and showed improvement from baseline in the atrophy grade at the lesser curvature of gastric corpus.^25^ In addition, a recent nationwide cohort study in Korea observed a lower GC risk (HR = 0.88, 95% CI: 0.80–0.96) and a decreased metachronous gastric neoplasm development (HR = 0.76, 95% CI: 0.70–0.82) following *H. pylori* treatment among patients who received endoscopic resection for gastric dysplasia.^26^ Pooling this evidence with other relative RCTs,^23^^,^ ^24^ Ford et al. reported an RR of 0.49 (95% CI: 0.34–0.70) for *H. pylori* eradication associated with the risk of future GC in individuals with gastric neoplasia undergoing endoscopic mucosal resection.^29^

**Table 2:** Studies of *H. pylori* treatment versus placebo or no treatment in metachronous GC prevention in patients with gastric neoplasia undergoing endoscopic mucosal resection.

Study	Location	Study type	Last point of follow-up, years	*H. pylori* eradication therapy regimen	Eradication rate^a^, %	Mean age at baseline, years (range)	Sample size (GC cases)		Risk ratio (95% CI)
							Treatment	Placebo/control	
Fukase 2008^23^	51 hospitals in Japan	RCT	3	Lansoprazole 30 mg, amoxicillin 750 mg and clarithromycin 200 mg b.i.d. for 1 week.	74.9	69 (median, 20–79)	272 (9)	272 (24)	0.35 (0.16–0.78)
Choi 2018^a,^^24^	One hospital in Seoul, South Korea	RCT	6	Omeprazole 20 mg, amoxicillin 1 g and clarithromycin 500 mg b.i.d. for 1 week.	81.3	60 (20–75)	437 (18)	440 (36)	2.02 (1.14–3.56)^b^
Choi 2018^b,^^25^	One hospital in Goyang, South Korea	RCT	5.9	Rabeprazole 10 mg, amoxicillin 1 g, and clarithromycin 500 mg b.i.d. for 1 week.	80.4	60 (18–75)	194 (14)	202 (27)	0.50 (0.26–0.94)
Yoo 2023^26^	Nationwide in Korea	Cohort study	NR, median 5.6	Standard triple therapy or bismuth-based quadruple therapy.	NR	62 (>18)	33,747 (901)	68,831 (2441)	0.76 (0.70–0.82)

Abbreviations: b.i.d., two times per day; CI, confidence interval; GC, gastric cancer; *H. pylori, Helicobacter pylori*; NR, not reported; RCT, randomized controlled trial; t.i.d., three times per day.

a True intention-to-treat analysis, with all drop-outs assumed to have failed eradication therapy.

b Eradication group as reference in Choi 2018a.

## 3 Systemic consequences after *H. pylori* eradication treatment

While *H. pylori* eradication has been proven to have preventive effect on GC, the broader health implications of *H. pylori* eradication remain poorly understood. This lack of clarity poses a significant barrier to the broader application of *H. pylori* eradication across large populations. Here, we summarize evidence on the short- and long-term impacts of *H. pylori* eradication on the gastrointestinal microbiome and the hosts’ metabolic profiles, two key indicators of systemic health, to systematically elucidate the therapy's health impacts and assess its feasibility for population-wide application.

### 3.1 Changes of microbiota structure

Antibiotic treatment for *H. pylori* infection has been shown to impact gastric microbes, resulting in significant changes in microbial composition and diversity.^35^ Our team's prospective study based on the Linqu high-risk area using paired gastric biopsies and stool samples found that a 10-day course of quadruple therapy not only enhanced the diversity of gastric microbes and mitigated dysbiosis six months post-treatment, but also significantly increased gastric microbial richness and evenness in successful eradication cases, leading to the restoration of gastric microbiota with beneficial effects such as probiotic enrichment and downregulation of drug-resistant mechanisms.^36^ Beyond the significant reduction of *H. pylori* levels after treatment, several studies observed a notable rise in the relative abundances of other genera in the gastric microbiota, including *Prevotella, Neisseria, Pseudomonadaceae*, and *Enterobacteriaceae*, over both short- (≤6 months) and long-term (>6 months) follow-ups.^36^, ^37^, ^38^ Additionally, a recent retrospective study showed that *H. pylori*-positive subjects who underwent eradication therapy exhibited a reduced gastric microbial co-occurrence, which was characterized by a distinct cluster of oral bacteria including *Peptostreptococcus, Parvimonas, Fusobacterium, Haemophilus, Neisseria, Gemella, Granulicatella, Rothia, Streptococcus,* and *Porphyromonas*.^38^ Besides, an enrichment of *Acinetobacter lwoffii, Streptococcus anginosus*, and *Ralstonia* and a depletion of *Roseburia* and *Sphingomonas* were observed in those with persistent inflammation one year after treatment, contrasting with subjects who received placebo treatment and showed largely unchanged gastric microbial ecology.^38^ These findings together highlight a complex interplay between the potential consequences of *H. pylori* treatment and gastric microbiological environment.

*H. pylori* eradication has also been reported to induce notable alterations in the composition and balance of gut microbiota,^39^, ^40^, ^41^, ^42^ leading to both short-term changes and potentially long-lasting effects on its composition and function.^43^ Jakobsson et al. underscored the profound impact of short-term antibiotic use on gastrointestinal flora, with certain alterations in colonic microbiota lasting up to four years post-treatment.^44^ Subsequent studies have corroborated these findings, illustrating both short-term and long-term modifications in the gut microbiota following *H. pylori* eradication, predominantly using triple or bismuth quadruple therapy.^36^^,^^42^^,^^45^, ^46^, ^47^, ^48^, ^49^, ^50^, ^51^, ^52^, ^53^ While immediate post-treatment effects showed considerable shifts in gut microbiota diversity and composition, long-term impacts varied, with some studies indicating a recovery in gut microbial diversity,^44^^,^^46^^,^^48^^,^^51^^,^^54^ whereas others documented altered alpha diversity (the mean species diversity within a particular area or ecosystem).^45^^,^^47^ A recent meta-analysis examining variations in gut microbiota taxa across short- and long-term follow-up periods post-treatment highlighted specific genus-level fluctuations and varying responses among bacterial populations such as *Actinobacteria, Proteobacteria, Enterobacteriaceae*, and *Enterococcus*, underscoring the dynamics of microbial communities post-eradication therapy.^55^

Both the short- and long-term post-treatment alterations of gastric and gut microbiota suggest that treating *H. pylori* infection with antibiotics can unintentionally disturb the microbial balance.^56^ The disruption may persist, compromising vital gut functions, including the ability to prevent infection,^57^ regulate metabolism,^58^ and maintain mucosal immune homeostasis,^59^ and may alter the genomic capacity of gut microbial communities, leading to the selection and enrichment of resistant strain and species.^60^ Given the complex relationship between antibiotic treatments and microbial ecosystems, while a restoration of microbial diversity and beneficial shifts in microbial populations can be achieved following treatment, other persistent changes and drug-resistant microbes may emerge, thus emphasizing the critical need for strategies that mitigate adverse effects while enhancing the therapeutic outcomes of *H. pylori* eradication. For instance, it may be important to incorporate appropriate probiotics and prebiotics into treatment regimens to support microbiome balance recovery.^42^^,^^61^ Moreover, further research is critical to elucidate potential consequences via monitoring long-term changes in microbial ecology post-eradication^62^ and to develop therapeutic approaches that minimize the impacts on non-targeted bacteria.^63^

### 3.2 Impacts on metabolic profiles

Metabolism is related to the regulation of physiological and biological processes, with its disturbances closely associated with the onset and progression of tumors and other chronic diseases. Evidence has shown that *H. pylori* infection disrupts gastrointestinal flora, leading to metabolic abnormalities at the cellular level.^64^^,^^65^ Eradication of *H. pylori* has been reported to influence insulin sensitivity, lipid metabolism, and glucose metabolism. Specifically, the eradication of *H. pylori* leads to a decrease of pro-inflammatory cytokines induced by the infection, such as Tumor Necrosis Factor-α, Interleukin-6, and C-reactive protein,^66^ which interfere with insulin signaling and lipid metabolism.^67^^,^^68^ This may result in a reduction in systemic inflammation and mitigate the negative impact of the infection on metabolic functions, leading to improvements in insulin sensitivity and reductions in dyslipidemia.^69^ Moreover, *H. pylori* eradication can normalize gastric hormones such as ghrelin and leptin, which are crucial for maintaining energy homeostasis, and can further support lipid metabolism improvements by increasing high-density lipoprotein cholesterol levels.^70^, ^71^, ^72^ Considering the biological interplay between *H. pylori* eradication and metabolic traits, as well as the important role of the metabolic profiles in maintaining health balance, a comprehensive understanding of how *H. pylori* eradication affects the body's metabolic profile is essential for fully elucidating its full spectrum of health outcomes.

Several metabolomics studies have explored the relationship between *H. pylori* eradication and subsequent changes in host metabolic profiles. Fang et al. examined serum metabolite levels in *H. pylori*-positive children before and four weeks after eradication treatment, and observed decreases in trimethylamine N-oxide and creatine levels, as well as increases in lactate and low-density/very low-density lipoprotein levels post-treatment, indicating metabolic alterations related to energy, amino acid, lipid, and microbial metabolism.^73^ Orihara et al. revealed that successful *H. pylori* eradication in males significantly altered the fatty acid composition of gastric mucosal phosphatidylcholine six months after treatment, with increasing linoleic acid and decreasing arachidonic acid levels in the gastric antrum and body, respectively.^74^ Another study using fecal and blood samples of 29 Malaysian subjects analyzed by targeted and untargeted metabolomics showed that *H. pylori* eradication affects the host energy and lipid metabolism, which are associated with the development of metabolic disorders.^75^ Furthermore, a recent metabolomics study identified 59 metabolites with significant changes before and six months after treatment, 17 of which could discriminate successful eradication from failed treatment. These metabolites primarily involve glycerophospholipid and linoleic acid metabolism pathways.^76^

While the short-term impacts on metabolic profiles following *H. pylori* eradication have been widely documented, the long-term (over six months after eradication) effects on metabolic health have not been fully elucidated. Yap et al. reported a general decrease in plasma metabolites and lipid levels 18 months after treatment, yet this observation is tempered by a relatively small sample size (*n* = 12) during the 18-month follow-up.^75^ Wu et al.'s secondary analysis of an intervention trial (*n* = 186), with up to three years of follow-up post-treatment, revealed that *H. pylori* eradication significantly influenced short-term plasma metabolic profiles but seemed to have minimal long-term effects on metabolic regulation.^76^ Furthermore, a recent multi-centered, open-label, randomized trial highlighted that *H. pylori* eradication may bring improved metabolic parameters one year after treatment, including decreased insulin resistance, reduced triglycerides and low-density lipoprotein levels, and increased high-density lipoprotein, thus supporting the metabolic benefits of *H. pylori* eradication therapy.^47^ All these findings suggest that beyond the immediate goal of infection clearance, the treatment may have broader and long-term implications for metabolic health and disease risk management. The variability in study designs, follow-up periods, and metabolic biomarkers highlights the need for studies with adequate statistical power, robust validation, and multiple prospective follow-ups to fully elucidate the long-term metabolic outcomes of *H. pylori* eradication.

## 4 Practices of *H. pylori* test-and-treat strategy in real-world settings

The *H. pylori* test-and-treat strategy focuses on detecting the presence of *H. pylori* and subsequently eradicating it when detected.^77^ In addition to treating *H. pylori* symptomatic individuals, there has been international interest for the strategy in using *H. pylori* testing to screen asymptomatic individuals in order to prevent GC.^78^ In some regions, targeted *H. pylori* screening as part of the test-and-treat strategy is recommended for asymptomatic individuals at high risk, such as those with certain ethnic or geographic backgrounds associated with a high risk of GC.^79^, ^80^, ^81^, ^82^ In real-world settings, optimizing the management of *H. pylori*-positive individuals involves accurate *H. pylori* screening tests that are appropriate for detecting active infections. The three non-invasive methods that can be used for the test-and-treat strategy are serology, the ^13^C-UBT, and the stool antigen test. While some serology tests have demonstrated high sensitivity and specificity, their performance may vary by geographic location due to strain variation, highlighting the importance of validating tests locally to ensure accuracy and reliability across different regions.^83^^,^ ^84^ Generally, serology has been considered less accurate than the ^13^C-UBT and monoclonal stool antigen tests.^85^, ^86^, ^87^, ^88^ The ^13^C-UBT has been shown as the best approach to diagnosing *H. pylori* infection due to its high accuracy and ease of use.^89^ Stool antigen testing, while potentially less acceptable to individuals in some cultures, has been equally valid with high sensitivity and specificity when a monoclonal antibody-based enzyme-linked immunosorbent assay is used.^90^ In addition to non-invasive testing, the management of *H. pylori*-positive individuals should include endoscopic examinations for those at risk of GC. This approach ensures early detection and treatment of individuals at risk of progressing from early gastric lesions to high-grade intraepithelial neoplasia or GC, enabling timely intervention and playing a crucial role in the secondary prevention of GC.

## 5 Cost-effectiveness of *H. pylori* eradication for gastric cancer prevention at population level

Increasing health economic evaluations have suggested that screening of *H. pylori* infection followed by eradication treatment is cost-effective to prevent GC at the population level. This approach demonstrates dual benefits in both saving lives and achieving significant economic savings in healthcare across diverse geographical regions, which advocates the 'test-and-treat' strategy for *H. pylori* infection as an important approach to preventing GC. In Asian regions, the 'test-and-treat' strategy for *H. pylori* eradication is consistently acknowledged as cost-effective, showing a remarkably low incremental cost-effectiveness ratio (ICERs) of $1100 per Life Years Gained (LYG) and $24 per Quality-Adjusted Life Year (QALY) and outperforming the option of no screening.^91^, ^92^, ^93^, ^94^ Moreover, a nationwide *H. pylori* eradication strategy in Japan significantly lowered healthcare costs and reduced GC incidence and mortality across all age groups, yielding estimated savings of US$3.75 billion from 2013 to 2019—more than tenfold of the country's annual cancer control budget, underscoring its economic benefits and the importance of long-term follow-up.^95^ However, such economic benefits vary in Western countries. For instance, Teng et al. underscored the strategy's economic feasibility for the Māori population in New Zealand, pointing to its broader applicability.^96^ Further supporting this approach, Beresniak et al. found that the 'test-and-treat' strategy not only efficiently manages dyspepsia but also appears to be cost-effective in the prevention of ulcers and GC in Spain.^97^ Unlike these countries, in the United States, the strategy's cost-effectiveness for GC prevention varies across different demographic groups and depends on specific thresholds of cancer risk reduction,^63^ with ICERs ranging from a low of $4500/LYG in Japanese Americans to a high of $34,900/LYG in non-Hispanic White Americans.^63^^,^^98^, ^99^, ^100^ The variability in ICERs among the Western studies is largely attributed to differences in the estimates that are influenced by factors such as *H. pylori* prevalence, eradication therapy effectiveness, and the costs of testing and treatment.^98^^,^^99^^,^^101^, ^102^, ^103^

In China, the 'test-and-treat' strategy for *H. pylori* eradication has demonstrated considerable promise in preventing GC, showing significant benefits for both high-risk and asymptomatic populations and highlighting its potential for broad application and cost savings.^96^^,^^104^, ^105^, ^106^, ^107^ Zheng et al. identified that treating *H. pylori* was a cost-saving measure, offering better results at a lower cost particularly for close relatives of GC patients.^106^ Han et al. conducted a simulation study on Chinese populations, demonstrating that the implementation of *H. pylori* screening followed by eradication treatment significantly lowered both the occurrence of GC and the associated expenses in asymptomatic individuals.^104^ Furthermore, Chen et al. demonstrated that a population-wide approach involving screening and treating *H. pylori* was more cost-effective and efficient in preventing GC in the general asymptomatic population than a strategy without screening in China.^105^

Essential considerations for improving the economic benefits of the ‘test-and-treat' strategy remain a critical area of investigation. Recent Chinese studies reported that *H. pylori* screening every three and five years offers greater cost-efficiency compared to annual *H. pylori* screening,^108^ and beginning eradication efforts at age 20 could enhance both health outcomes and economic savings.^91^ Meanwhile, findings from the U.S. advocate for the cost-effectiveness of targeting individuals aged 40 with a familial risk of GC.^109^ The success of the ‘test-and-treat’ strategy also depends on the balance between the diagnostic accuracy and costs,^110^ with economic evaluations favoring the stool antigen test and UBT for their cost-effectiveness in *H. pylori* screening.^100^^,^^111^ Further studies highlight UBT's economic advantages in GC prevention,^103^ especially in high-risk or older populations, emphasizing the need for adaptable screening and treatment protocols that reflect varied demographic and economic contexts.

## 6 Impact of antibiotic resistance and host characteristics on eradication failure

Among factors such as individual non-adherence, obesity, and unhealthy lifestyles that may diminish the success of the anti-*H. pylori* therapy,^27^^,^^112^, ^113^, ^114^, ^115^ antibiotic resistance is the most important cause of *H. pylori* eradication failure. A systematic review highlighted geographical variations in *H. pylori* antibiotic resistance, reporting global resistance rates for clarithromycin (17.2%), metronidazole (26.7%), levofloxacin (16.2%), amoxicillin (11.2%), tetracycline (5.9%), rifabutin (1.4%), and multiple antibiotics (9.6%), with resistance to clarithromycin, metronidazole, and levofloxacin specifically increasing from Europe to Asia, America, and Africa.^116^ Meanwhile, a prospective multi-region study on Chinese patients found *H. pylori* strains showing the highest resistance to metronidazole (67.2%), followed by clarithromycin (37.5%), levofloxacin (33.5%), rifampicin (14.2%), amoxicillin (6.8%), and tetracycline (3.5%), with resistance ranging from mono-resistance (34.2%) to sextuple-resistance (0.3%), influenced by sex, age (for levofloxacin), and peptic-ulcer or non-ulcer diseases visible under endoscopy (for clarithromycin, metronidazole, and levofloxacin).^117^ The current largest trial on *H. pylori* eradication for GC prevention, MITS, reported a relatively successful community-based treatment strategy with a 72.9% elimination rate using tetracycline and metronidazole, with an estimated combined resistance rate of 5.32% to these antibiotics, determined using the E-test method on cultures from biopsy samples of 153 participants from the pilot study of MITS.^27^

The recent global rise in antibiotic resistance, particularly for clarithromycin, poses a significant challenge to the effectiveness of standard therapies for *H. pylori* treatment. For instance, the prevalence of clarithromycin resistance has surged from 3% to 11% around the turn of the century and currently standing at 15–30% globally.^118^, ^119^, ^120^ A recent meta-analysis integrating findings from 178 studies revealed that the prevalence of primary (i.e. for treatment-naïve individuals) and secondary (i.e. for previously treated individuals) resistance rates to clarithromycin, metronidazole, and levofloxacin was over 15% in all regions.^120^ Moreover, a significant association was identified between clarithromycin resistance and the failure of clarithromycin-containing regimens (OR = 6.97, 95% CI: 5.23–9.20).^120^ The World Health Organization has designated clarithromycin-resistant *H. pylori* infections as a significant concern among community-acquired infections,^121^ and international guidelines recommend abandoning clarithromycin-based regimens if regional resistance surpasses 15%.^112^ In challenges of clarithromycin resistance, antibiotic susceptible testing (AST) before initiating *H. pylori* treatment, a strategy explored in several RCTs, was recommended by several consensus reports.^122^^,^^123^ Despite so, a recent meta-analysis found no significant difference in eradication rates between treatments guided by AST and those based on empirical therapeutic strategies.^124^ Beyond clarithromycin resistance, the rapid development of high resistance rates to levofloxacin poses another threat,^119^ given its crucial role in rescue regimens.^125^^,^^126^ Additionally, metronidazole has a consistently stable resistance rate over 25% in most areas of the world,^120^ yet its resistance's negative impact on treatment outcomes is mitigated by the synergistic compensation of co-administered drugs.^127^^,^^128^ Besides, no relevant resistance issues have been identified with amoxicillin, tetracycline, and rifabutin.

Even though individuals might confirm antibiotic susceptibility and patient adherence, eradication failure still occurs.^129^ Accordingly, additional host characteristics contributing to eradication failure have been explored. Research indicates that *H. pylori* eradication success is influenced by multiple factors, with smoking and drinking habits consistently being significant contributors to treatment failure in men,^114^^,^^115^^,^^130^ probably due to their effects on gastric acid secretion and antibiotic delivery.^114^^,^^115^^,^^130^ The MITS highlights the role of high baseline delta over baseline (DOB) values as a predictor of eradication failure, suggesting that patients with high bacterial loads may benefit from extended therapies (duration from 10 to 14 days).^27^ Additionally, a grade-response relationship between BMI and eradication failure in women was observed in MITS, which implies that those with higher BMI might also benefit from extended therapies.^27^ In spite of high treatment compliance, the MITS further identified that missed doses emerged as a critical factor for eradication failure, especially in women and those with higher education levels who may miss doses due to busy daily work.^27^ Apart from behavioral factors, genetic factors such as *CYP2C19* and *IL1B* polymorphisms also play crucial roles in *H. pylori* eradication success,^131^ affecting drug metabolism and gastric acid suppression, and thus significantly influencing individuals’ treatment outcomes.

## 7 Other consequences of *H. pylori* eradication that may raise attention

Other than GC prevention, the benefits of *H. pylori* eradication have been documented for dyspepsia,^132^ peptic ulcer disease (PUD),^133^ and gastric mucosa-associated lymphoid tissue (MALT)lymphoma.^134^^,^^135^ As suggested by the Maastricht VI/Florence consensus report, a test-and-treat strategy for *H. pylori* infection is suitable for uninvestigated dyspepsia in populations with a high prevalence of infection (≥20%).^122^ In addition, the eradication therapy has been shown to improve symptoms in H. pylori-positive functional dyspepsia,^132^ heal duodenal ulcers better than ulcer-healing drugs,^136^ and reduce gastrointestinal bleeding in older patients on aspirin.^133^ For MALT lymphoma, eradicating H. pylori leads to complete histological remission in most patients with localized MALT lymphoma.^134^^,^^137^ The ACG clinical guideline suggests that patients with active PUD, a history of PUD (unless previously cured of *H. pylori* infection), or low-grade gastric MALT lymphoma should receive *H. pylori* eradication therapy.^123^ *H. pylori* infection has also been identified as a potential causal factor in colorectal carcinogenesis,^138^ emphasizing the need for integrating *H. pylori* status into colorectal cancer (CRC) preventive measures. In line with this understanding, a recent retrospective study among US veterans assessed the impact of *H. pylori* infection and its treatment on CRC incidence and mortality, revealing that *H. pylori* infection is positively associated with both CRC incidence and mortality, and treatment for the infection is associated with reductions in these risks.^139^

Besides, *H. pylori* infection is commonly acquired in early childhood and is associated with health challenges such as iron deficiency, growth impairment, and malabsorption, indicating complex implications and alternative consequences of its eradication on early childhood health.^140^ Evidence from interventional trials illustrates that *H. pylori* eradication significantly improves iron deficiency and iron deficiency anemia,^141^, ^142^, ^143^, ^144^, ^145^, ^146^, ^147^ enhancing hemoglobin and/or ferritin levels,^141^, ^142^, ^143^ particularly when combined with oral iron supplementation.^148^ However, findings from Bangladesh and observations in malaria-endemic regions reveal a nuanced picture: While eradication benefits iron status, it may increase malaria risk,^149^ thus necessitating careful consideration for treatment implementation in such areas.^140^^,^^150^ Additionally, *H. pylori* treatment has been reported to show positive effects on children's growth in Colombia and China, with treated children showing notable height and weight gains.^151^^,^^152^ This evidence highlights the bacterium's influence on childhood development but also leaves the research gap in understanding its effects in the first two years of life. Moreover, *H. pylori* treatment in infants and young children with hypochlorhydria has been linked to health improvements and reduced mortality,^140^^,^^153^^,^^154^ illustrating the potential for better nutrient absorption through restored gastric acid secretion and altered intestinal microbiota.

**Fig. 2:**
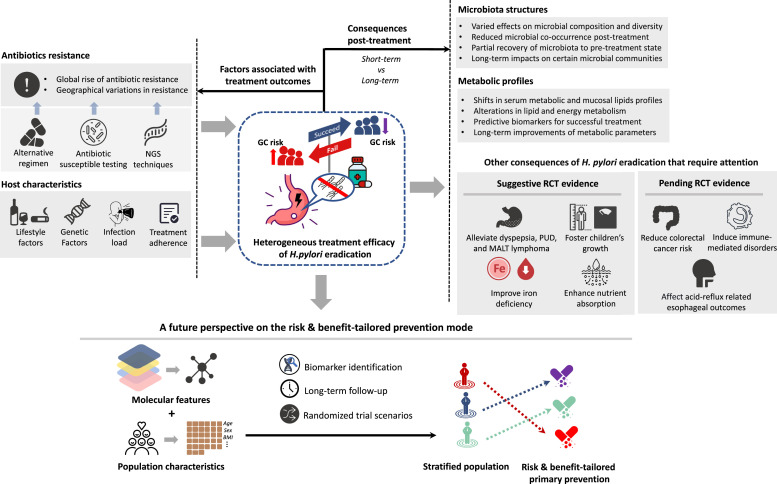
Integrated overview of *H. pylori* eradication and gastric cancer prevention strategies. The figure summarizes the dynamic interactions between antibiotic resistance and host factors that influence the outcomes of *H. pylori* eradication in the context of gastric cancer prevention, underscoring the transition to risk & benefit-tailored prevention strategies. The interplay of evolving antibiotic resistance patterns and individual patient characteristics contributes to a spectrum of treatment consequences, from shifts in microbiota structure to metabolic alterations, along with other divergent post-treatment effects. DOB, delta over baseline; GC, gastric cancer; *H. pylori*, Helicobacter pylori; MALT, mucosa-associated lymphoid tissue; NGS, next-generation sequencing; PUD, peptic ulcer disease.

Albeit the potential health benefits of *H. pylori* eradication, it is worth noting that unaddressed concerns still exist regarding the possibly adverse effects of *H. pylori* treatment. In addition, studies are required to further elucidate the impact of *H. pylori* treatment on esophageal adenocarcinoma^31^^,^^155^ and other acid-reflux-related esophageal outcomes such as Barrett's esophagus and gastroesophageal reflux,^155^, ^156^, ^157^ for which inconsistent associations with *H. pylori* infection have been reported. These findings collectively suggest that *H. pylori* eradication can have significant health benefits but also highlight the importance of a balanced approach considering the potential for unintended consequences (Fig. 2).

## 8 Challenges and directions

Despite the already well-established causality of *H. pylori* triggering gastric carcinogenesis and recognized beneficial effect of *H. pylori* treatment for GC prevention, most infected individuals do not develop GC during their lifetime. For infected individuals deemed with successful eradication, many still develop GC during follow-up, without displaying long-term benefits of *H. pylori* treatment.^20^ These concur with the complexity of GC etiology, which involves the interplays of genetic susceptibility and environmental factors with *H. pylori* infection.^158^

Whether eradication would be particularly effective in specific environmentally exposed subgroups is also of translational implications to clarify.^159^ Population-level research has demonstrated the effectiveness and efficacy of *H. pylori* eradication in preventing GC in high-risk areas, as evidenced by follow-up data from our community-based intervention trials.^20^ Moreover, families with shared lifestyles can be considered as an important factor in defining exposed subgroups for intervention, given *H. pylori*’s features of family cluster infection and intrafamilial transmission.^160^ The novel concept of ‘whole family-based *H. pylori* infection control and management’ has been reported as effective and convenient in clinical practice due to its better engagement of family members, higher eradication rates, lower reinfection rates, and cost-effectiveness,^160^^,^^161^ and was recommended by the *Helicobacter pylori* Study Group of Chinese Society of Gastroenterology.^162^

Recent studies have indicated that integrating multi-layered individual-level data, particularly through advancements in molecular and multi-omics insights, can provide valuable insights for identifying the target population for GC prevention. Emerging multi-omics and data science technologies offer researchers an avenue to fully consider the complexity of GC etiology and the impact of multidimensional factors on the effectiveness of primary prevention at the research level. Multi-omics research provides an opportunity to identify potential key molecular biomarkers, allowing us to develop candidate strategies and simple, practical testing tools. These tools may help integrate environmental, genetic, and host factors to predict effectiveness and evaluate risk, guiding the selection of candidate populations for *H. pylori* eradication in primary prevention and optimizing prevention and control strategies. For instance, our team's recent prospective study found that *H. pylori* treatment particularly benefited a high genetic risk subgroup population (those with top quartile of the polygenic risk score) in GC prevention, partly suggesting that primary prevention could be tailored with genetic risk for more effective prevention. The pinpointed genetic factors are closely related to the signaling pathways activated by *H. pylori* virulence factor CagA, providing insights into the heterogeneous treatment efficacy in GC prevention.^163^ Apart from this, ongoing efforts such as the *Helicobacter pylori* Genome Project, which focuses on elucidating *H. pylori* pathogenesis and identifying new therapeutic targets across diverse *H. pylori* and human populations, also support multi-omics integration as a valuable perspective for addressing *H. pylori* treatment heterogeneity in GC prevention.^164^

Once the target population is identified, it would be crucial to tackle the drug resistance problem that limits individuals’ chances of having successful eradication outcomes. While the societal consequences of the massive use of antibiotics should also be taken into account,^31^ future directions to combat *H. pylori* antibiotic resistance in the primary prevention of GC emphasize developing new antimicrobials and AST tools, with next-generation sequencing (NGS) offering promising avenues for non-invasive susceptibility testing and understanding resistance mechanisms.^165^^,^^166^ Besides, information regarding the host-specific determinants of eradication outcomes should be incorporated and validated in real-world settings by leveraging large-scale population cohort resources. For instance, NGS enables the analysis of the bacterial 'resistome' using multi-omics approaches and supports large-scale population surveillance,^165^^,^^167^ providing valuable feedback and new insights for the optimization of existing evaluation systems. Moreover, NGS-based metagenomics can generate complete bacterial genomes directly from samples, providing an alternative to culture-based methods and enhancing insights into the mechanisms of antibiotic resistance.^166^^,^^168^^,^^169^

## 9 Conclusions

Comprehensive population interventions can significantly reduce the disease burden of *H. pylori* and prevent GC, offering cost-effectiveness advantages. The existing evidence underscores the efficacy and effectiveness of *H. pylori* test-and-treat in preventing GC, yet its implications extend beyond infection clearance, necessitating a deeper understanding of the treatment's multifaceted consequences. Despite the cost-effectiveness of a population-wide test-and-treat strategy, the potential limitations of this approach, along with the complexities of GC development, highlight the importance of targeting high-risk individuals and subgroups for preventive strategies. It is also worth noting that a “one-size-fits-all” approach to eradicating *H. pylori* infection may neither be biologically plausible nor practically efficient. Along with *H. pylori* treatment, other strategies may be useful for the primary prevention of GC as well.^20^^,^^34^ With multidisciplinary efforts comprehensively elucidating the environmental risk factors and molecular events underlying gastric carcinogenesis, we will be able to construct integrated models for risk assessment and effect evaluation of both primary and secondary prevention strategies, establishing risk and effect-tailored prevention and management modes of GC.

## Declaration of completing interest

The authors declare that they have no known competing financial interests or personal relationships that could have appeared to influence the work reported in this paper.
